# Equipment Capability Measurement of Laboratory Freeze-Dryers: a Comparison of Two Methods

**DOI:** 10.1208/s12249-021-01921-2

**Published:** 2021-01-19

**Authors:** Jayasree M. Srinivasan, Gregory A. Sacha, Vaibhav Kshirsagar, Alina Alexeenko, Steven L. Nail

**Affiliations:** 1 Pharmaceutical R&D, Baxter Healthcare, 927 S. Curry Pike, Bloomington, Indiana 47403 USA; 2 Present Address: IMA Life, Inc., Tonawanda, New York 14150 USA; 3 School of Aeronautics and Astronautics, Purdue University, West Lafayette, Indiana 47907 USA; 4 Davidson School of Chemical Engineering, Purdue University, West Lafayette, Indiana 47907 USA

**Keywords:** equipment capability, minimum controllable pressure, choke point, choked flow, tunable diode laser absorption spectroscopy (TDLAS)

## Abstract

The objective of this investigation was to evaluate two methods for measuring the maximum sublimation rate that a freeze-dryer will support—the minimum controllable pressure method and the choke point method. Both methods gave equivalent results, but the minimum controllable pressure method is preferred, since it is easier, faster, and less subjective. The ratio of chamber pressure to condenser pressure corresponding to the onset of choked flow was considerably higher in this investigation (up to about 20:1) than in previously published reports. This ratio was not affected by the location of the pressure gauge on the condenser; that is, on the foreline of the vacuum pump *versus* on the body of the condenser itself. The total water loss due to sublimation as measured by tunable diode laser absorption spectroscopy was consistently within 5% of gravimetrically determined weight loss, regardless of whether the measurement took place during choked *versus* non-choked process conditions.

## INTRODUCTION

Freeze-drying is an essential unit operation in manufacture of injectable drug products, but the process suffers from being very inefficient, with cycle times of days rather than a few hours, as in most pharmaceutical unit operations. The inherent inefficiency of the process is commonly made worse by a trial-and-error approach to cycle development, where process conditions may be developed that are far from optimum, where optimum conditions are defined as those that minimize cycle time while providing a pharmaceutically acceptable product using conditions that are within the capability of the equipment.

**Fig. 1:**
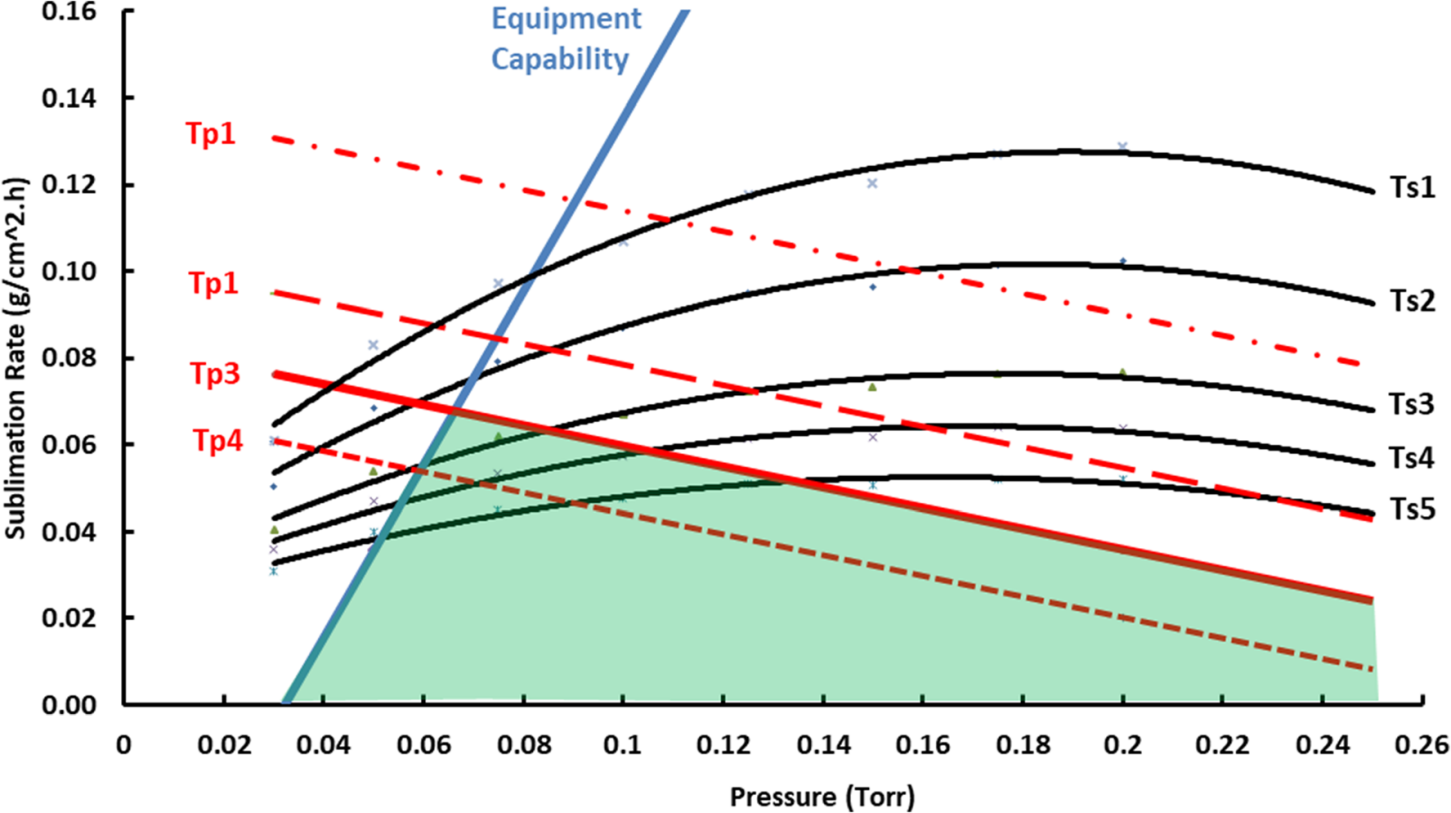
Representative design space indicating equipment capability curve (blue trace) as one of two boundaries. Red dashed lines indicate product temperature isotherms. Red solid line indicates critical product temperature isotherm. All black traces indicate shelf temperature isotherms

Identification of optimum conditions for primary drying has been facilitated in recent years by application of a graphical design space for primary drying (1,2). A representative design space is illustrated in Fig. 1, with sublimation rate on the *y*-axis and chamber pressure on the *x*-axis. There are two sets of isotherms—one for shelf temperature and one for product temperature. The design space is constructed using first principles of heat and mass transfer along with measured values for the vial heat transfer coefficient (*K*_v_) and the resistance of the dried product layer to flow of water vapor during primary drying (*R*_p_). These isotherms establish the relationship between the process variables that are directly controlled, shelf temperature and chamber pressure, and product temperature, a critical process variable that is not directly controlled.

There are two boundaries on the design space in Fig. 1—one product-related and the other equipment-related. One of the product temperature isotherms (solid red line) represents the maximum allowable product temperature during primary drying (usually, but not always, the collapse temperature). The other boundary (blue trace) is the equipment capability curve. This curve recognizes that any freeze-dryer has limits with respect to the maximum sublimation rate that it will support. Limiting factors could be refrigeration capacity, condenser surface area, or maximum attainable shelf temperature. However, for many freeze-dryers using an external condenser design, equipment capability is limited by sonic velocity in the duct connecting the freeze-dryer chamber to the condenser (3). Sonic velocity is the maximum obtainable flow velocity for a constant cross-section pipe. As water vapor velocity approaches the speed of sound (about 390 m/s at − 25°C), the mass flow rate through the duct attains its maximum value for a given upstream (chamber) pressure and becomes independent of pressure on the downstream side of the duct—in this case, the condenser pressure. This is known as choked flow. Note, in Fig. 1, that the maximum sublimation rate is a linear function of chamber pressure.

Equipment limitations imposed by choked flow were identified by Searles as a source of uncertainty in scale-up (3). This is particularly important for formulations that are very robust under aggressive drying conditions, either because of a high upper product temperature limit during primary drying, a low resistance of the dry product layer to flow of water vapor, or both. A valuable lesson from Searles’s work is that cycle conditions need to be developed with an eye toward the capability of the equipment to be used for commercial production.

The purpose of this article is to compare two procedures for measuring equipment capability, namely, the minimum controllable pressure method and the choke point method, and to identify the method of choice. Secondary objectives were to determine whether the location of the pressure gauge on the condenser affects the chamber-to-condenser pressure ratio at the onset of choked flow, and to measure the quantitative accuracy of the TDLAS instrumentation under conditions of choked flow as well as under non-choked conditions. LyoStar III freeze-dryers were used for the measurements, but the methods described here should be applicable to any freeze-dryer with an external condenser design and a cylindrical duct connecting the product chamber and the condenser. TDLAS capability is very helpful, but not essential, to apply these methods.

## MATERIALS AND METHODS

Both test methods require the use of ice slabs on every shelf of the freeze-dryer. Three lyophilization tray frames (without the tray bottom) were lined with a black plastic sheet (an ordinary trash bag), and the edges of the plastic sheet were held in place using binder clips. The trays were loaded on to the shelves of the freeze-dryer in a three-shelf configuration and filled with 2 l of Milli-Q water each. The water was then frozen overnight at a shelf temperature of − 45°C.

Two different LyoStar 3® freeze-dryers (SP Scientific, Gardiner, NY) were used, where the only difference between the two units was the location of the capacitance manometer pressure gauge on the condenser. On one unit, the gauge is located on the foreline of the vacuum pump, very close to the condenser. On the other, the pressure gauge is connected to the body of the condenser at the approximate mid-point of the condenser front-to-back. The reason for using two different dryers was to determine whether the location of the condenser pressure gauge has an influence on the chamber-to-condenser pressure ratio at the onset of choked flow. Chamber pressure was measured by a capacitance manometer at the top of the chamber, in the back, for both freeze-dryers used. The duct dimensions for the LyoStar III freeze-dryers are listed in Table I.

**Table I:** Duct Dimensions for a LyoStar III Freeze-Dryer

Parameter	Dimension
Process path (cm)	13.1
Duct angle (deg)	45
Duct area (m^2^)	74.36
Duct radius (m)	0.0486
Duct distance (m)	0.12

Tunable diode laser absorption spectroscopy (TDLAS, Lyo-Flux 200, Physical Sciences, Inc., Andover, MA) was used as an in-process mass flow meter for water vapor (4). TDLAS is a near infrared (NIR)-based technology where the laser is tuned to detect water vapor absorption to measure the density (molecules/cc) of water vapor as well as its velocity (m/s). The optical hardware is installed in the duct that connects the freeze-dryer chamber and the condenser. TDLAS can only be used with dryers that are equipped with an external condenser, where the chamber and condenser are connected by a cylindrical duct, and the duct can accommodate the TDLAS optical hardware. The freeze-dryer/TDLAS used for this investigation is an integrated system that provides the following advantages over the previous stand-alone versions: (1) automated determination of the TDLAS sensor zero offset velocity; (2) automated saving of the TDLAS sensor data and integration of the total amount of water removed; (3) transfer of the TDLAS sensor data to the freeze-dryer data historian, providing consistent time stamps for the TDLAS and the freeze-dryer process data; (4) transfer of freeze-dryer chamber pressure and shelf temperature data to the TDLAS sensor automating data analysis for recipes with changing freeze-dryer operating pressure and enabling automated calculation of data products from the heat and mass transfer model of vial-based pharmaceutical freeze-drying; and (5) elimination of the need for an operator to be present at the start of a cycle or when a change in pressure set point is needed.

### Minimum Controllable Pressure Method

For the minimum controllable pressure method, the chamber pressure set point was 10 mT, which is below the lowest attainable pressure for the freeze-dryers under consideration. The initial shelf temperature was − 45°C. After a steady state chamber pressure was reached, the mass flow rate of water vapor was recorded. The shelf temperature was then increased by 10°C, and the process was repeated for several more shelf temperature set points. While only two points are required to establish a straight line, we consider it best practice to collect data for five or six pressures. With the minimum controllable pressure method, flow was choked throughout the experiment.

### Choke Point Method

After freezing the trays of water at − 45°C overnight, the system was evacuated and the pressure was allowed to stabilize at 45 mTorr. When steady state was established at this pressure set point, step changes to the shelf temperature were made until choked flow was observed. The resulting mass flow rate was then recorded. A new pressure set point was then established and the process was repeated.

### TDLAS Accuracy Measurement

For both test methods, the test was stopped when roughly 60–75% of the original ice load remained. The remaining ice was then weighed, and the percent error in the TDLAS measurement was calculated. For the choke point method, flow is choked during only part of the test, but the oscillations in both pressure and mass flow rate (see below) would appear to present a more challenging situation for accurate mass flow rate measurement by TDLAS.

For comparative data on TDLAS accuracy under entirely non-choked conditions, ice slab tests were done under modest process conditions, using a shelf temperature of either − 15°C or 0°C and a chamber pressure of 100 mT.

## RESULTS AND DISCUSSION

### Minimum Controllable Pressure Method

**Fig. 2:**
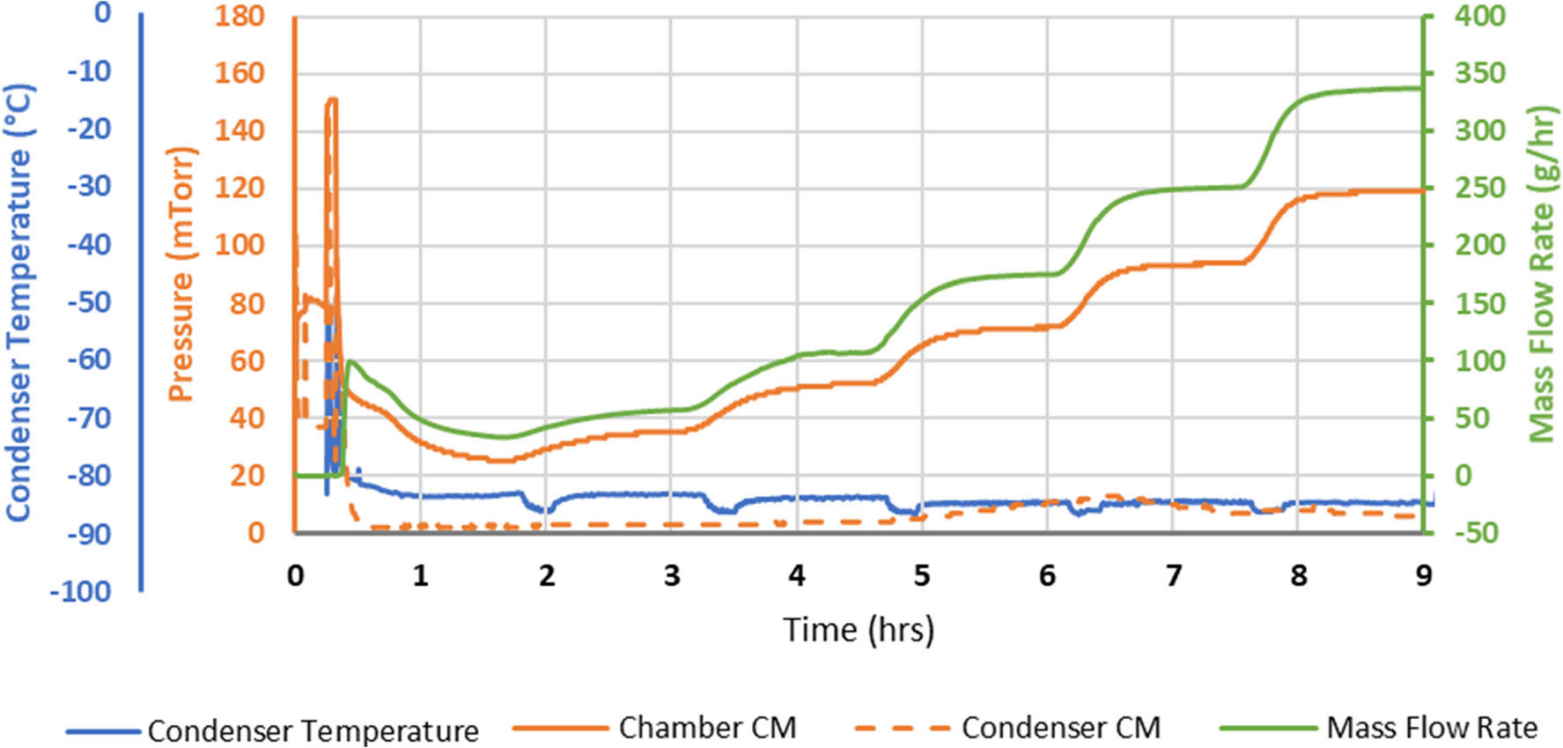
Graph of pressure and mass flow rate as a function of time using the minimum controllable pressure method. Note that the condenser temperature remained at < − 70°C throughout the experiment

The lyo in-process data were used to graph the pressure and the mass flow rate as a function of time (Fig. 2). The condenser pressure ranged from 2 to 13 mTorr throughout the experiment. As a result, the ratio of chamber pressure to condenser pressure ranged from approximately 8:1 to as high as 20:1. This is a surprisingly high pressure ratio as compared with literature reports. Searles (3) reported the use of the ratio of chamber pressure to condenser pressure as an indicator of choked flow, where a ratio of three or more should be taken as confirmatory of choked flow. Isentropic flow theory (5) gives the theoretical pressure ratio between the upstream and downstream reservoirs that correspond to choked conditions as *p*/p* = ((γ + 1)/2)^γ/(γ + 1)^ where γ is the specific heat ratio (*C*_p_/*C*_v_), *p* is the chamber pressure, and p* is the pressure at the duct exit. For water vapor at low temperatures, γ = 1.33 and *p*/p* = 1.85. A chamber-to-condenser ratio of less than 1.85 is taken as confirmation that the flow is not choked. Due to viscous effects not accounted for in the isentropic flow theory, the actual pressure ratio at which choking occurs for a given freeze-dryer may be higher than 1.85 and is highly dependent on the equipment design. There is some uncertainty regarding the pressure at the duct exit, with the condenser pressure gauge located either on the foreline of the vacuum pump, near the condenser body, or on the condenser itself on the top of the condenser roughly midway along the length. Given that water vapor is constantly being removed by the condenser (condenser temperature usually in the range of − 80 to − 85°C), we would expect pressure gradients in the condenser.

Another factor that may affect the chamber-to-condenser pressure ratio corresponding to the onset of choked flow is the presence of isolation valve hardware inside the duct. The theoretical pressure ratio assumes a “clean” cylinder. Kshirsagar and coworkers (6) reported that the isolation valve, contained within the connecting duct in the LyoStar freeze-dryers, reduces the cross-sectional area available for flow of gasses. The resulting pressure drop causes an increase in chamber pressure and would increase the chamber-to-condenser pressure ratio. These investigators did not attempt to calculate the expected pressure ratio under choked conditions. Patel *et al.* (7), using choke point testing, reported that the chamber-to-condenser pressure ratio was always greater than 2 when control of chamber pressure was lost.

In our experiment, the mass flow rate increased linearly with chamber pressure as the shelf temperature was increased in increments of 10°C. Note that the condenser temperature was unaffected by choked flow and remained at less than − 75°C throughout (Fig. 2). For the equipment used here, condenser performance is not a limiting factor in equipment capability.

### Choke Point Method

**Fig. 3:**
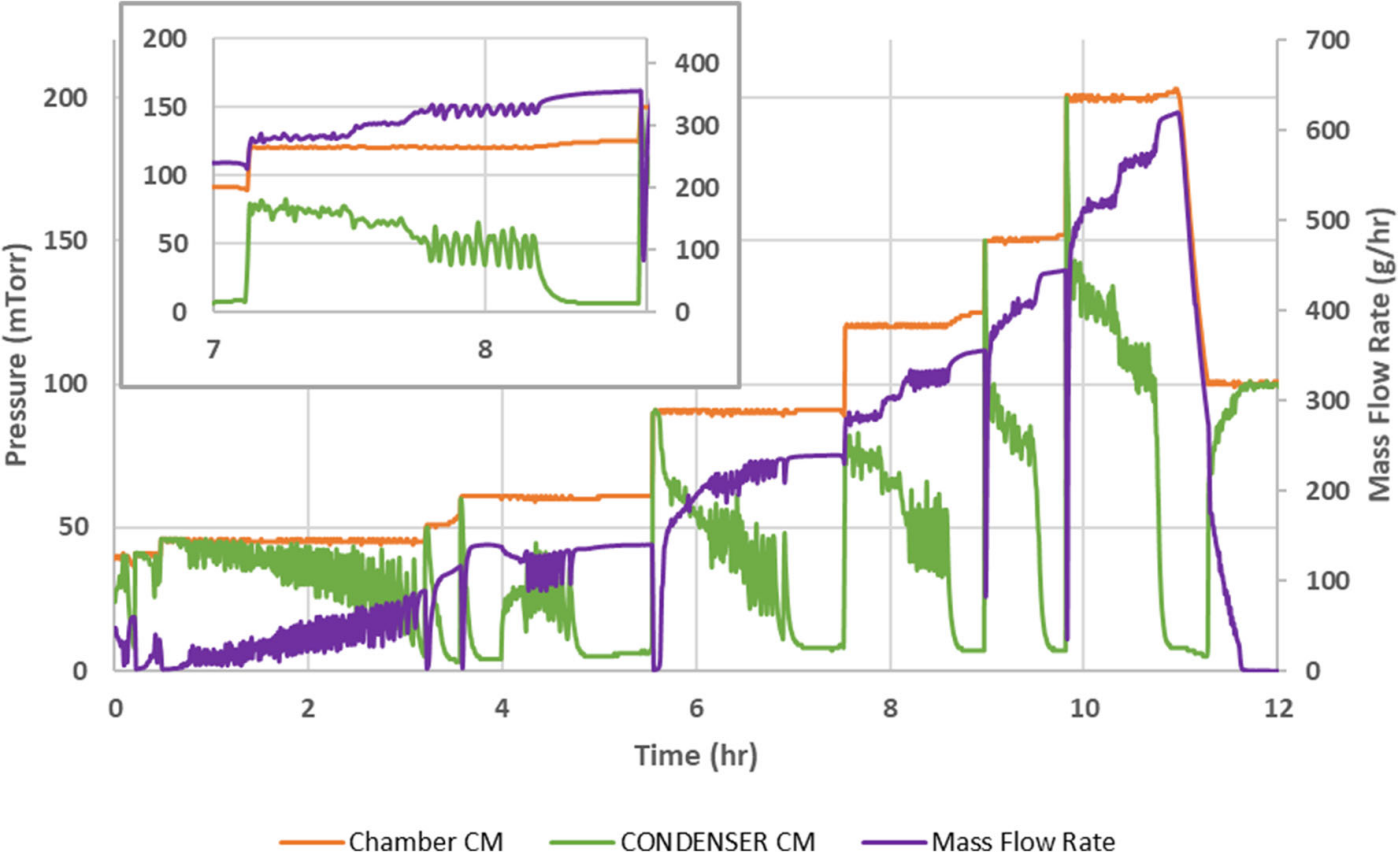
Graph of pressure and mass flow rate as a function of time using the choke point method and expanded graph to show condenser pressure “bottoming out” when the flow is choked at a pressure set point of 120 mTorr (inset)

Referring to the process data in Fig. 3, note that there are two ways of identifying choked flow using this method. One way is to observe that the chamber pressure drifts above the set point pressure. With respect to the process data in Fig. 3, this is a subtle change that might easily be overlooked. While chamber pressure exceeding the set point is evident at a set point of 120 mT (inset, at about 8.7 h), there are some set points where the pressure exceeding the set point is hardly evident at all. A more telling indicator of choked flow is the condenser pressure. Note that there is a period where both the mass flow rate and the condenser pressure are oscillating out-of-phase as the control system tries to maintain the chamber pressure set point. During this period of oscillation, as condenser pressure decreases, mass flow rate increases, and vice versa. By definition, flow of water vapor is not choked at this point, despite a chamber/condenser pressure ratio in the range of 2 to 3. At the point where the chamber pressure exceeds the set point, note that the condenser pressure drops rapidly. This is the point where the flow of nitrogen, used to control the pressure, cuts off, and the condenser pressure “bottoms out” at less than 10 mT, indicating the onset of choked flow. This probably corresponds to the low pressure limit of the vacuum system. These data demonstrate the utility of having a capacitance manometer on both the chamber and the condenser, particularly as a tool in measuring equipment performance.

### Effect of Condenser Pressure Gauge Placement

The data reported here used the freeze-dryer with the condenser pressure gauge placed on the foreline of the vacuum pump. However, we found that a minimum controllable pressure test using the freeze-dryer with the condenser pressure gauge mounted on the body of the condenser resulted in process data that were not distinguishable from the data in Fig. 2 (data not shown). These data support the idea that, at least for the two condenser pressure gauge configurations shown here, the minimum controllable pressure results are unaffected by the location of the pressure gauge. Of course, it would be useful to examine the influence of placing the pressure gauge at the duct exit.

### Comparison of the Two Methods

**Fig. 4:**
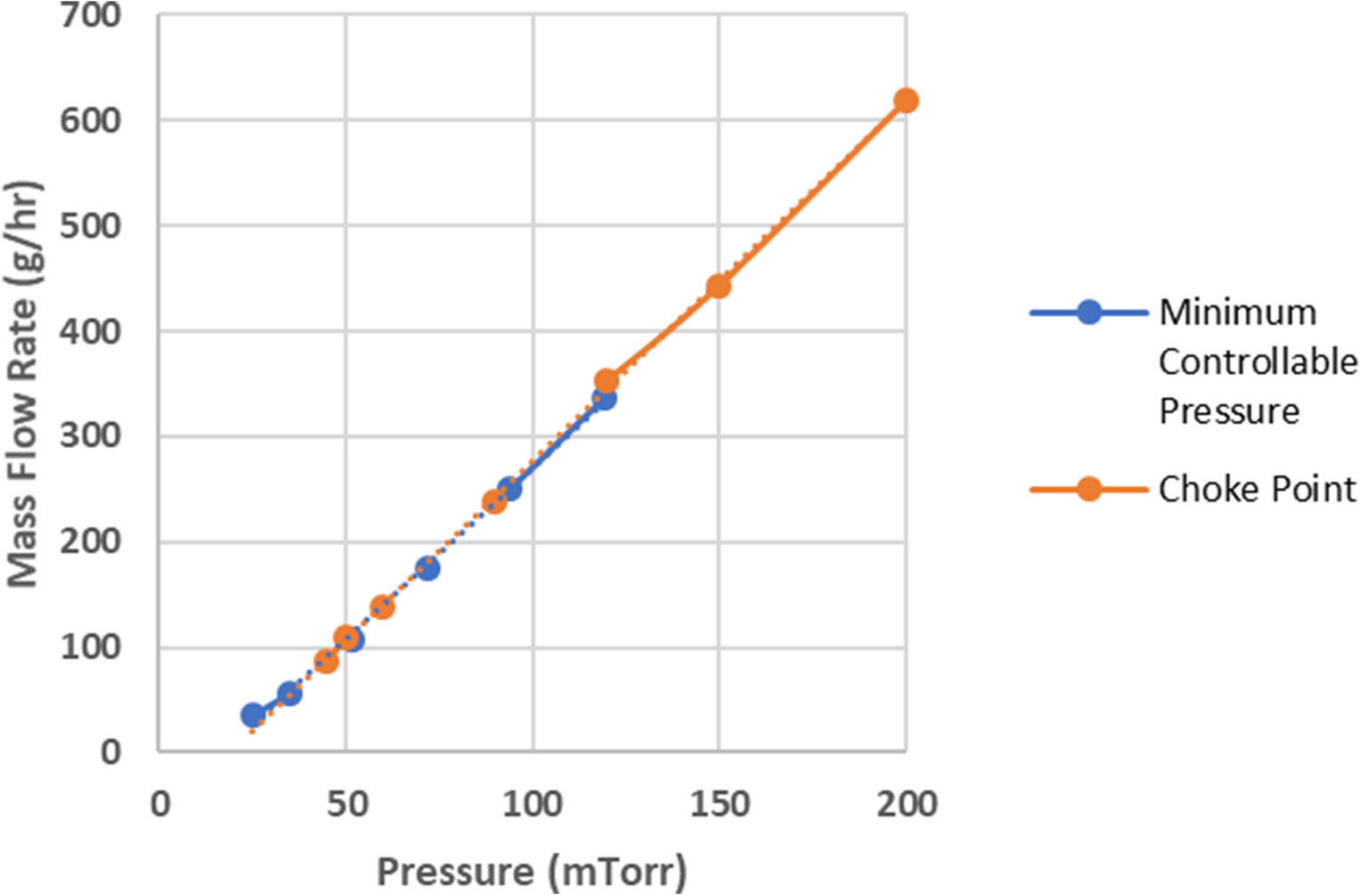
Comparison of minimum controllable pressure and choke point methods

Comparison of the mass flow data obtained using the minimum controllable pressure and choke point methods showed an excellent agreement between the two methods, as indicated by the superimposable lines in Fig. 4. Based on the results of this study, we recommend the minimum controllable pressure method because it is easier, quicker, and less subjective than the choke point method. In fact, the choke point method would not be advisable at all without the additional information provided by a pressure gauge on the condenser, given the often uncertainty around the point where the chamber pressure has exceeded the set point. Also, the minimum controllable pressure method allows for the experiment to be conducted in a fully automatic mode, whereas the choke point method allows for only a semi-automatic cycle with user intervention required periodically to determine the onset of choked flow.

### TDLAS Accuracy

The accuracy of the TDLAS as a flow meter to measure the mass flow rate was determined by comparing the quantity of ice sublimed as measured by TDLAS with gravimetric weight loss for several ice slab tests. The experiments were conducted either at steady state under non-choked conditions, when sublimation occurred at 0°C or − 15°C using a chamber pressure of 100 mTorr (Table II, runs 1–3), during minimum controllable pressure testing under choked flow (Table II, run 4), and during choke point testing (Table II, run 5). The TDLAS results were within 5.5% of the gravimetric results, indicating the accuracy of the TDLAS as an in-process flow meter. Also worth noting is that the resolution of the top loader balance that was used for the gravimetric weights is 10 g which introduces a source of uncertainty with the gravimetric weights. Our results are consistent with those reported by Patel and coworkers (7) who determined that the accuracy of TDLAS was unaffected by whether flow was choked or not.

**Table II:** Comparison of Gravimetric and TDLAS Weight Losses During Ice-Slab Experiments

Run #	Choked or non-choked flow	Gravimetric weight loss (g)	TDLAS weight loss (g)	TDLAS error (%)
1	Non-choked	820	865	5.5
2	Non-choked	5380	5143	− 4.4
3	Non-choked	2200	2290	4.1
4	Choked	2820	2930	3.9
5	Choked	2800	2840	1.4

## CONCLUSION

The equipment capability curve of a LyoStar III freeze-dryer was measured using two test methods: (a) minimum controllable pressure and (b) choke point. The results obtained using both methods were equivalent. However, the minimum controllable pressure method required less time to conduct the testing and hence is the recommended method by the authors.
